# Clinical significance of the expression of connexin26 in colorectal cancer

**DOI:** 10.1186/1756-9966-29-79

**Published:** 2010-06-21

**Authors:** Shinya Nomura, Kiyoshi Maeda, Eiji Noda, Toru Inoue, Shinya Fukunaga, Hisashi Nagahara, Kosei Hirakawa

**Affiliations:** 1 Department of Surgical Oncology, Osaka City University Graduate School of Medicine, 1-5-7 Asahimachi Abeno-ku, Osaka, 545-8585, Japan

## Abstract

**Background:**

Connexin26 (Cx26) is one of the connexins (Cxs) family members which form gap junction channels. Cx26 is considered to be a tumor suppressor gene. However, recent studies revealed that over expression of Cx26 is associated with a poor prognosis in several human cancers. This study investigated the correlation between Cx26 expression and the clinicopathological features and P53 expression in colorectal cancer.

**Methods:**

One hundred and fifty-three patients who underwent a curative resection were studied. Tissue samples were investigated by immunohistochemical staining using antibodies for Cx26 and P53. Moreover, apoptotic cells were detected by terminal deoxynucleotidyl transferase-mediated dUTP-biotin nick end-labeling (TUNEL) staining.

**Results:**

Cx26 expression was found in 83 cases (54.2%) and P53 expression in 71 cases (46.4%). A correlation was observed between the Cx26 expression and recurrence, histology, and p53 expression (P < 0.05). Cx26 positive tumors had significantly longer survival than Cx26 negative tumors (P < 0.05). A multivariate Cox analysis demonstrated that Cx26 expression was an independent prognostic factor (P < 0.05). However, no significant correlation was observed between Cx26 and AI.

**Conclusion:**

This study suggests that Cx26 expression is an independent prognostic factor in patients that undergo a curative resection of colorectal cancer.

## Introduction

A gap junction is a specialized intercellular connection that directly connects the cytoplasm of two cells, and allows various molecules and ions ( < 1 kDa) to pass freely between cells. Gap junctional intercellular communication (GJIC) mediated by gap junctions play an important role in regulating homeostasis, proliferation and differentiation [1,2]. Gap junction channels contain two hemichannels that are primarily homo -or hetero-hexamers of connexin (Cx) proteins [3]. Twenty types of Cx have been identified as transmembrane proteins [4]. A reduction or loss of GJIC function associated with human carcinomas such as skin cancer, lung cancer, gastric cancer, hepatocellular carcinoma, glioma and prostate cancer, is usually induced by down-regulation of Cxs [5-9]. Moreover, restoration of GJIC in tumor cell lines by Cx transfection can reduce growth and tumorigenicity [10,11]. Therefore, Cxs are thought to be tumor suppressor genes. However, over expression of Cx26 might the acquisition of malignant phenotypes and is correlated with metastasis, tumor grade and prognosis in several carcinomas [12-14]. Therefore, this study examined the correlation between Cx26 expression by immunohistochemistry in colorectal carcinoma and clinicopathological features and P53 expression as a tumor suppressor gene.

## Materials and methods

This study evaluated 153 patients with colorectal carcinoma who underwent a curative resection at the Department of Surgical Oncology (First Department of Surgery) of Osaka City University Graduate School of Medicine (Osaka, Japan). The age of the patients ranged 30 from 84 years (mean 65.5 years); and there were 87 males and 66 females were included. All of them underwent a curative resection and were followed for at least 5 years after surgery. Hematoxylin and eosin-stained slides were reviewed and the diagnoses were confirmed.

Tumor staging was defined according to the criteria for histological classification proposed by the International Union Against Cancer (UICC). Patients were informed of the investigational nature of the study and each provided written informed consent prior to recruitment.

Resected specimens from these patients were fixed in a 10% formaldehyde solution and embedded in paraffin. Four micrometer thick sections were cut and mounted on glass slides.

### Immunohistochemical method

Cx26 and P53 immunostaining were performed by the streptavidin-biotin method. As primary antibodies, mouse monoclonal anti-Cx26 (Zymed Laboratories, San Francisco, CA, working dilution 1:500) and mouse monoclonal P53 antibodies (DAKO, Carpinteria, CA, ready to use) were used. The sections were cut (4 μm), dried for 4 h at 58˚C, and then dewaxed in xylene and dehydrated through an ethanol series. Endogenous peroxidase was blocked by incubation with 0.3% H2O2 in methanol for 30 min at room temperature. Thereafter, the sections were autoclaved for 10 min at 121˚C in 10 mM sodium citrate (pH 6.0). The sections were washed with phosphate-buffered saline (PBS) and incubated with 10% normal rabbit serum for 10 min to reduce non-specific staining. The specimens were incubated with the respective primary antibodies in a moist chamber overnight at 4°C. The specimens were washed with PBS and incubated in a secondary antibody for 10 min at room temperature. The sections were washed three times in PBS and incubated with the streptavidin-peroxidase reagent for 5 min at room temperature. Finally, the sections were incubated for 5 min in PBS containing diaminobenzidine and 1% hydrogen peroxide (Histofine SAB-PO kit, Nichirei), followed by counterstaining with Mayer's hematoxylin. As the negative control, incubation with the primary antibody was omitted.

Moreover, we investigated the apoptotic cells by terminal deoxynucleotidyl transferase-mediated dUTP-biotin nick end-labeling (TUNEL) staining, using an In Situ Apoptosis Detection Kit (MK-500; Takara bio Co., Tokyo, Japan) according to the manufacturer's instructions.

### Staining Analysis

**Figure 1:**
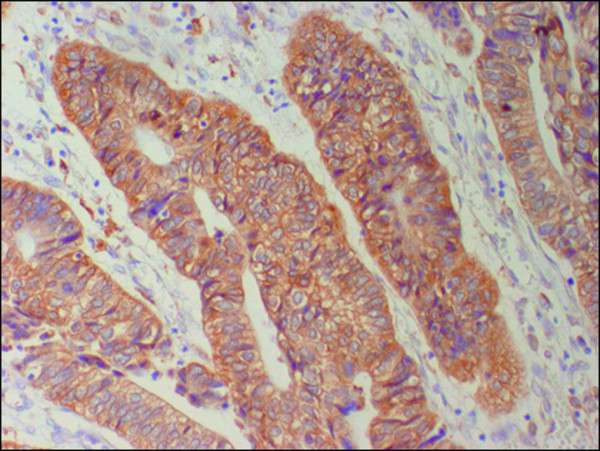
**Immunohistochemical staing for Cx26 in colorectal cancer. Cytoplasmic Cx26 expression was found (×200)**.

**Figure 2:**
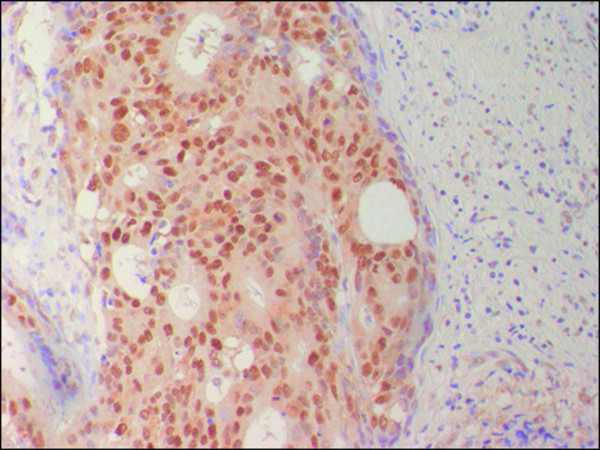
I**mmunohistochemical staing for P53 in colorectal cancer.** Nuclear P53 expression was found in most tumor cells (×200).

**Figure 3:**
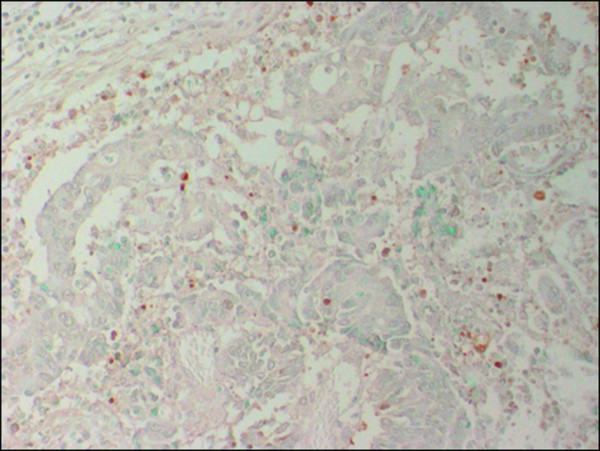
**Apoptotic index (AI) as evaluated by TUNEL (×200)**.

Immunoreactivity for Cx26 was considered to be positive if distinct staining of the cytoplasm was observed in at least 10% of the tumor cells (Fig. 1) and P53 was considered to be positive if distinct staining of the nuclei was observed in at least 50% of tumor cells (Fig. 2). The apoptotic index (AI) was expressed as the number of apoptotic tumor cells divided by the total number of tumor cells in the same field with evaluation of 1000 nuclei in randomly selected areas in each specimen (Fig. 3).

The slides were examined by two independent pathologists who were not aware of the corresponding clinicopathological data. Any cases with discordant scores were reevaluated a second time until a consensus was reached, no discrepancies between the evaluations were detected by the two investigators.

### Statistical Analysis

The data were compiled and analyzed using the SPSS software package for Windows (version 11.0; SPSS Inc., Chicago, Ill., USA). The relationship between Cx26 expression and the clinicopathological data, P53 and AI was evaluated by the chi-square test and Mann-Whitney U test. The disease specific survival was calculated by the Kaplan-Meier method and analyzed by the log-rank test. Prognostic factors were examined by univariate and multivariate analyses using a Cox proportional hazards model. P < 0.05 was considered to be significant.

## Results

**Figure 4:**
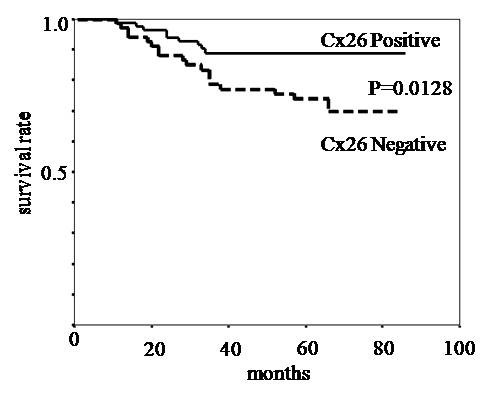
**The disease specific survival according to Cx26 expression**. Patients with Cx26 positive tumors showed significantly longer survival than those with Cx26 negative tumors (P = 0.0128)

**Figure 5:**
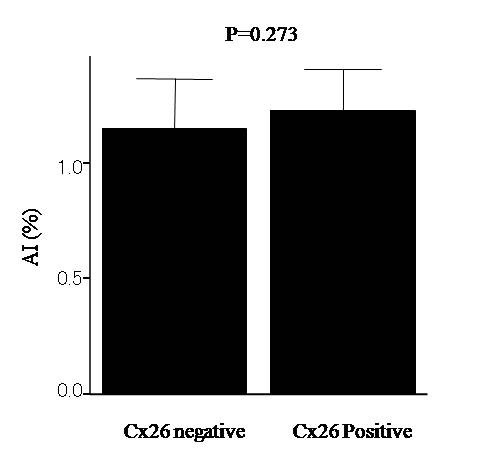
**Value of apoptotic index (AI) according to Cx26 expression**. No significant correlation was found (P = 0.273)

Cx26 expression was mainly localized in the cytoplasm of the cancer cells. In a few cases, we observed weak cytoplasmic staining in the normal mucosa. However we did not consider this to be specific staining. Eighty-three of the 153 tumors (54.2%) showed Cx26 expression. P53 expression was observed in 71 (46.4%). The correlation between Cx26 and the clinicopathological features is summarized in Table 1. Cx26 expression had a statistically significant relationship with disease recurrence and the histological type (P < 0.05). Moreover P53 expression had a statistically significant relationship with Cx26 expression (P < 0.05). The disease specific survival according to the status of Cx26 expression is shown in Fig.4. The patients with Cx26 negative tumors had significantly worse survival than those with positive tumors (P < 0.05). Cx26 expression was an independent prognostic factor, as well as lymph node metastasis, blood vessels invasion according to a multivariate analysis (Table 2). There was no significant correlation between Cx26 and AI (Fig. 5).

**Table 1:** Correlation between the Cx26 expression and clinicopathological features

	Cx26		
		
	Negative	Positive	P-value
Age (mean ± SD, years.	66.4 ± 8.1	66.4 ± 10.5	
Gender			
Male	41	46	
Female	29	37	0.695
Tumor size (mean ± SD, cm.	5.0 ± 2.1	5.1 ± 2.9	
Lymphatic invasion			
Negative	24	25	
Positive	46	58	0.582
Blood vessel invasion			
Negative	60	68	
Positive	10	15	0.528
Lymph node metastasis			
Negative	47	53	
Positive	23	30	0.670
Site			
Colon	47	60	
Rectum	23	23	0.489
Depth of invasion			
~mp	17	11	
~ss	53	72	0.079
Disease recurrence			
Negative	44	65	
Positive	26	18	0.035
Histological type			
Well	22	27	
Moderately	37	55	
Others	11	1	0.003
P53			
Negative	31	51	
Positive	39	32	0.034

**Table 2:** Univariate and multivariate survival analyses of the prognostic factors

Multivariate analysis				
Variable	Comparsiion	Hazard ratio	P-value	95% CI
Cx26	Negative : Positive	3.734	0.002	1.607-8.674
Lymph node metastasis	Positive : Negative	2.587	0.027	1.115-5.999
Lymphatic invasion	Positive : Negative	2.584	0.139	0.735-9.083
Vessel invasion	Positive : Negative	4.084	0.002	1.687-9.887
Tumor size	>5 cm : ≦5 cm	2.658	0.065	0.941-7.507
Univariate analysis				
Cx26	Negative : Positive	2.651	0.017	1.191-5.903
Lymph node metastasis	Positive : Negative	4.720	<0.001	2.118-10.516
Lymphatic invasion	Positive : Negative	4.387	0.016	1.320-14.580
Vessel invasion	Positive : Negative	4.044	<0.001	1.844-8.870
Tumor size	>5 cm : ≦5 cm	3.961	0.005	1.500-10.462

## Discussion

Several studies of colorectal carcinoma reported that Cx26 expression is found mainly in the plasma membrane in normal epithelium and malignant transformation is associated with the loss of plasma membrane staining and increased cytoplasmic staining [15-18]. However, Knösel et al. also reported the Cx26 expression to be observed in the cytoplasm of colon cancer cells, while it was not observed in the normal mucosa [19]. Our current data showed the same results. The Cx26 expression was observed in the cytoplasm in 54.2% of the colorectal tumors in the current series. Although, the mechanism of cytoplasmic staining was unclear, we therefore assumed the cytoplasmic staining of Cx26 to be independent from the GJIC- mechanism in colon cancer.

Several studies reported that Cx26 expression is associated with poor prognosis in lung and esophageal squamous cell carcinoma and breast cancer [13,14,20]. However Knösel et al. [19] reported that reduced Cx26 expression is significantly associated with shorter patients' survival and higher tumor grade. The current study also found that patients with Cx26 negative tumors had worse survival than those with Cx26 positive tumors. Moreover, the multivariate analysis showed that Cx26 was an independent prognostic factor.

Cx26 is thought to be a tumor suppressor gene, but mechanism which regulates tumor suppression is unclear. Several studies have reported suggestive evidence that the tumor-suppressive effects of Cx expression are GJIC-independent [10,21]. Cytoplasmic staining of Cx26 was considered to be a GJIC-independent mechanism. Cx26 may have an effect on other tumor related genes. Hong et al. reported a significant correlation between the Cx26 expression and P53 expression [17]. P53 is a common tumor suppressor gene and plays a major role in regulating the cell cycle and apoptosis [22]. The expression of P53 in colorectal cancer is thought to be associated with poor prognosis [23-25]. A mutation of the P53 is frequently observed in several human tumors. The expression of P53 protein is equivalent to the presence of a mutation of the p53 gene [26]. Therefore, we investigated the relationship between Cx26 and P53 protein. Cx26 expression had an inverse correlation with P53 expression. Cx26 positive tumors tended to have negative P53 expression.

On the other hand, p53 gene regulates apoptosis and P53 positive tumors show decreased AI [27]. Therefore, the relationship between Cx26 and AI was investigated. However, there was no significant relationship between Cx26 and AI.

In conclusion, the Cx26 function in cancer cells is unclear. Cx26 expression was an independent prognostic factor in colorectal cancer in the current series. Therefore, an analysis of the Cx26 expression may be useful for selecting patients who are at high risk for recurrence.

## Competing interests

The authors declare that they have no competing interests.

## Authors' contributions

EN and KM contributed to the conception and design of the study; SN, EN and KM contributed to collection and assembly of data; SN, EN, KM, TI, SF, HN and KH contributed to data analysis and interpretation; SN, KM, EN contributed to manuscript writing. All authors have read and approved the final manuscript.
